# Acknowledgments

**DOI:** 10.1002/ags3.12899

**Published:** 2025-01-01

The publication of invaluable papers in *Annals of Gastroenterological Surgery* depends on the prompt, careful review of submitted manuscripts. We would like to thank the following experts for reviewing manuscripts submitted between December 1, 2023 and November 30, 2024.

Reviewer Full Name

Abe, Tatsuya

Abe, Yuta

Aizawa, Masaki

Ajiki, Tetsuo

Akahoshi, Keiichi

Akamatsu, Nobuhisa

Akita, Hirofumi

Akiyoshi, Takashi

Aoki, Takeshi

Aoki, Taku

Aoyama, Toru

Araki, Kenichiro

Arigami, Takaaki

Arita, Junichi

Baba, Kenji

Ban, Daisuke

Bekki, Yuki

Booka, Eisuke

Daiko, Hiroyuki

Ebata, Tomoki

Ebihara, Yuma

Eguchi, Hidetoshi

Etoh, Tsuyoshi

Fujimura, Takashi

Fujita, Fumihiko

Fujita, Takeo

Fukagawa, Takeo

Fukami, Yasuyuki

Fukushima, Ryoji

Ganeko, Riki

Hagi, Takaomi

Hamabe, Atsushi

Hanaoka, Marie

Harada, Kazuto

Harimoto, Norifumi

Haruki, Koichiro

Hasegawa, Kiyoshi

Hasegawa, Yasushi

Hashimoto, Daisuke

Hashimoto, Masashi

Hatano, Etsuro

Hayami, Shinya

Hayashi, Hiromitsu

Hibi, Taizo

Hida, Koya

Hidaka, Masaaki

Hijioka, Susumu

Hirano, Satoshi

Hirano, Yasumitsu

Hirashita, Teijiro

Hiyoshi, Yukiharu

Honda, Goro

Hosoda, Kei

Ichikawa, Nobuki

Iguchi, Tomohiro

Ikeda, Masataka

Ikeda, Satoshi

Ikeuchi, Hiroki

Ikoma, Hisashi

Imamura, Yu

Inaki, Noriyuki

Inoue, Mikihiro

Iseda, Norifumi

Ishido, Keinosuke

Ishihara, Soichiro

Ishizawa, Takeaki

Ishizuka, Mitsuru

Itatani, Yoshiro

Ito, Takashi

Itoh, Shinji

Iwatsuki, Masaaki

Jiang, Xingming

Kagawa, Hiroyasu

Kagawa, Yoshinori

Kaibori, Masaki

Kaido, Toshimi

Kajiwara, Yoshiki

Kanda, Mitsuro

Kanemitsu, Yukihide

Kasai, Shunsuke

Kato, Atsushi

Kato, Motohiko

Kato, Yutaro

Kawachi, Shigeyuki

Kawada, Kenji

Kawai, Kazushige

Kawai, Manabu

Kawamura, Junichiro

Kawazoe, Tetsuro

Kim, Dong‐Sik

Kimura, Yasutoshi

Kimura, Yutaka

Kinami, Shinichi

Kinoshita, Takahiro

Kitagawa, Akihiro

Kitago, Minoru

Kitai, Toshiyuki

Kitayama, Joji

Kiyomatsu, Tomomichi

Kobayashi, Hirotoshi

Kobayashi, Minako

Kobayashi, Shogo

Kobayashi, Toshimichi

Koda, Keiji

Koike, Yuhki

Komatsu, Shuhei

Konishi, Hirotaka

Kosuga, Toshiyuki

Kosumi, Keisuke

Koyama, Fumikazu

Koyanagi, Kazuo

Kubo, Shoji

Kubota, Takeshi

Kumagai, Koshi

Kumagai, Youichi

Kunisaki, Chikara

Kuroda, Shinji

Lee, Sang‐Woong

Makino, Isamu

Manabe, Tatsuya

Marubashi, Shigeru

Maruyama, Suguru

Matsuda, Akihisa

Matsuda, Keiji

Matsuda, Kenji

Matsuhashi, Nobuhisa

Matsumoto, Ippei

Matsumoto, Yoshihiro

Matsuo, Yasuko

Matsuyama, Takatoshi

Mayanagi, Shuhei

Miki, Yuichiro

Mima, Kosuke

Mine, Shinji

Misawa, Takeyuki

Mise, Yoshihiro

Miura, Fumihiko

Miyamoto, Yuji

Miyo, Masaaki

Mizuno, Rei

Mizuno, Shugo

Mizuno, Takashi

Monden, Kazuteru

Morikane, Keita

Morikawa, Takanori

Morine, Yuji

Morise, Zenichi

Motoi, Fuyuhiko

Motomura, Takashi

Motoyama, Satoru

Mukohyama, Junko

Nagakawa, Yuichi

Nagase, Hayato

Naitoh, Takeshi

Nakada, Koji

Nakagawa, Kei

Nakajima, Kentaro

Nakamoto, Yuji

Nakamura, Toru

Nakamura, Yoshiharu

Nakase, Hiroshi

Nakata, Kohei

Nakayama, Izuma

Nakayama, Yusuke

Namikawa, Tsutomu

Niihara, Masahiro

Nishidate, Toshihiko

Nishino, Hitoe

Noma, Kazuhiro

Noshiro, Hirokazu

Nunobe, Souya

Oba, Takuya

Obama, Kazutaka

Ofuchi, Takashi

Ogura, Atsushi

Ohashi, Manabu

Ohira, Gaku

Ohira, Masahiro

Ohtsuka, Masayuki

Ohtsuka, Takao

Okabayashi, Koji

Okada, Ken‐ichi

Okamoto, Kohji

Okamoto, Koichi

Okamoto, Michio

Okamura, Ryosuke

Okamura, Yukiyasu

Okano, Keiichi

Okita, Yoshiki

Okuchi, Yoshihisa

Okugawa, Yoshinaga

Okumura, Shintaro

Okuno, Keisuke

Omura, Kenji

Orimo, Tatsuya

Oshima, Nobu

Ota, Mitsuhiko

Ouchi, Akira

Ozato, Yuki

Poudel, Saseem

Rikitake, Ryoko

Saito, Takuro

Saito, Yu

Sakamoto, Yoshihiro

Sato, Hiroshi

Satoi, Sohei

Seo, Satoru

Seto, Yasuyuki

Shibasaki, Susumu

Shibata, Chikashi

Shichinohe, Toshiaki

Shida, Dai

Shigeyasu, Kunitoshi

Shimada, Hideaki

Shimada, Mitsuo

Shimada, Yoshifumi

Shindoh, Junichi

Shinmoto, Hiroshi

Shinohara, Hisashi

Shinto, Eiji

Shiomi, Akio

Shiozaki, Atsushi

Shiraishi, Takuya

Shirakawa, Yasuhiro

Shoda, Katsutoshi

Soejima, Yuji

Sonoda, Hirofumi

Suda, Koichi

Sugimachi, Keishi

Sugiura, Teiichi

Suto, Hironobu

Suwa, Yusuke

Suzuki, Shuji

Takahara, Takeshi

Takahashi, Hidenori

Takahashi, Ryo

Takahashi, Takao

Takahashi, Tsuyoshi

Takasu, Chie

Takatsuki, Mitsuhisa

Takeishi, Kazuki

Taketomi, Akinobu

Tanaka, Koji

Tanaka, Kuniya

Tanaka, Naoki

Tanaka, Yoshihiro

Tani, Masaji

Thorsen, Kenneth

Tokumitsu, Yukio

Tokunaga, Masanori

Tomimaru, Yoshito

Tominaga, Tetsuro

Toshima, Takeo

Tsukamoto, Fumio

Tsukamoto, Shunsuke

Tsunoda, Shigeru

Tsurumaru, Daisuke

Uchida, Yoichiro

Uchino, Motoi

Uemura, Kenichiro

Uemura, Mamoru

Ueno, Masaki

Umeda, Yuzo

Ushigome, Hajime

Wada, Hidetoshi

Wakai, Toshifumi

Watanabe, Hiroaki

Watanabe, Kazuhiro

Watanabe, Manabu

Watanabe, Masayuki

Watters, David

Xiao, Miao

Yagi, Shintaro

Yamada, Masahiro

Yamada, Suguru

Yamada, Takeshi

Yamaguchi, Shigeki

Yamaguchi, Tomohiro

Yamamoto, Kazuyoshi

Yamamoto, Seiichiro

Yamamoto, Yusuke

Yamashita, Kotaro

Yamashita, Yo‐Ichi

Yanagimoto, Yoshitomo

Yano, Hideaki

Yasuda, Satoshi

Yasui, Masayoshi

Yoh, Tomoaki

Yokoo, Hideki

Yoshikawa, Takaki

Yoshitomi, Hideyuki

Yoshiya, Shohei

